# Genome Features and AntiSMASH Analysis of an Endophytic Strain *Fusarium* sp. R1

**DOI:** 10.3390/metabo12060521

**Published:** 2022-06-04

**Authors:** Yuanyuan Liu, Meijie Xu, Yuqi Tang, Yilan Shao, Hong Wang, Huawei Zhang

**Affiliations:** School of Pharmaceutical Sciences, Zhejiang University of Technology, Hangzhou 310014, China; liuyuan_0507@163.com (Y.L.); xumeijie1230@163.com (M.X.); t17670659912@163.com (Y.T.); shaoyilan1999@163.com (Y.S.); hongw@zjut.edu.cn (H.W.)

**Keywords:** endophytic fungus, *Fusarium*, whole-genome sequence, secondary metabolite, biosynthetic gene cluster, antiSMASH

## Abstract

Endophytic fungi are one of the most prolific sources of functional biomolecules with therapeutic potential. Besides playing an important role in serious plant diseases, *Fusarium* strains possess the powerful capability to produce a diverse array of bioactive secondary metabolites (SMs). In order to in-depth mine gene clusters for SM biosynthesis of the genus *Fusarium*, an endophytic strain *Fusarium* sp. R1 isolated from *Rumex madaio* Makino was extensively investigated by whole-genome sequencing and in-depth bioinformatic analysis, as well as antiSMASH annotation. The results displayed that strain R1 harbors a total of 51.8 Mb genome, which consists of 542 contigs with an N50 scaffold length of 3.21 Mb and 50.4% GC content. Meanwhile, 19,333 functional protein-coding genes, 338 tRNA and 111 rRNA were comprehensively predicted and highly annotated using various BLAST databases including non-redundant (Nr) protein sequence, nucleotide (Nt) sequence, Swiss-Prot, Gene Ontology (GO), Kyoto Encyclopedia of Genes and Genomes (KEGG) and Clusters of Orthologous Groups (COG), as well as Pathogen Host Interactions (PHI) and Carbohydrate-Active enzymes (CAZy) databases. Antibiotics and Secondary Metabolites Analysis Shell (AntiSMASH) results showed that strain R1 has 37 SM biosynthetic gene clusters (BGCs), including 17 nonribosomal peptide synthetases (NRPSs), 13 polyketide synthetases (PKSs), 3 terpene synthases (Ts), 3 hybrid NRPS + PKS and 1 hybrid indole + NRPS. These findings improve our knowledge of the molecular biology of the genus *Fusarium* and would promote the discovery of new bioactive SMs from strain R1 using gene mining strategies including gene knockout and heteroexpression.

## 1. Introduction

Endophytic fungi have been considered one of the richest sources of natural products with diverse chemical structures and biological properties, which play a potential role in the development of new therapeutical agents [1,2,3]. The landmark in this area of endophyte bioprospecting was undoubtedly the discovery of *Taxomyces andreanae*, the first taxol-producing endophytic fungus from *Taxus brevifolia* [4]. Since then, the secondary metabolites (SMs) of endophytic microbes have received a wide range of attention. Fungi belonging to the genus *Fusarium* are prevalent on crops in both semitropical and temperate zones since they can infect vegetables, fruits, small grain cereals and maize, leading to vascular wilt, stem, root, and ear rot, with a serious decrease in the yields of crops and severe economic losses [5,6]. However, a great amount of evidence indicates that the genus *Fusarium* possesses the potential capability to produce plenty of SMs with significant bioactivities, such as antimicrobial [7,8], anticancer [9,10], antiviral [11], antioxidants [12,13], and so on.

Genome mining is one computational method for the automatic detection and annotation of biosynthetic gene clusters (BGCs) from genomic data. During the past two decades, this approach has been increasingly utilized in natural product (NP) discovery due to the large amount of genome sequencing data that is now available [14]. As a comprehensive pipeline for the automated mining of genome data for the presence of BGCs, antiSMASH (antibiotics and Secondary Metabolites Analysis Shell) has made a significant contribution to microbial genome mining for novel SM discovery [15]. The SM reservoir of *Fusarium* species shows these strains harbor a wide array of BGCs including polyketide synthases (PKSs), non-ribosomal peptide synthetases (NRPSs), and terpene synthases (TSs), hybrids and miscellaneous, suggesting that its biosynthetic potential goes substantially beyond compounds commonly classified as “*Fusarium* toxins” [16].

However, the results of our previous research are not interesting or exciting since most of these SMs that we found are polyketide compounds and other types of chemicals such as terpenes have not yet been obtained from strain R1. It suggests that most SM BGCs in strain R1 are silent or expressed at a low level under conventional cultivation conditions. In order to in-depth explore the SM biosynthetic potential of strain R1, its whole-genome sequencing and analysis, as well as antiSMASH annotation are extensively conducted in this work.

## 2. Results and Discussion

### 2.1. Identification of Strain R1

**Figure 1:**
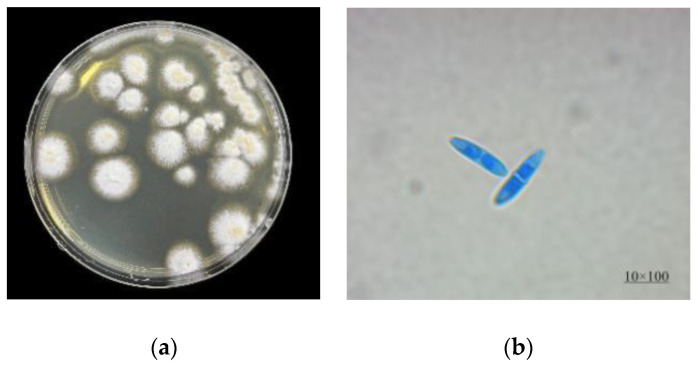
Colony (**a**) and microscopic (**b**) morphology of strain R1.

After being incubated on a PDA medium for 3 d at 28 °C, strain R1 produced white colonies with aerial mycelium and fusiform conidia (Figure 1). Phylogenetic analyses of the *18S* rRNA sequence (GenBank accession no. MF376147) and the ITS sequence [17,18] (GenBank accession no. ON545070) indicated that strain R1 was unambiguously determined as *Fusarium* species (Figures S1 and S2).

### 2.2. Genome Sequencing and Assembly

**Figure 2:**
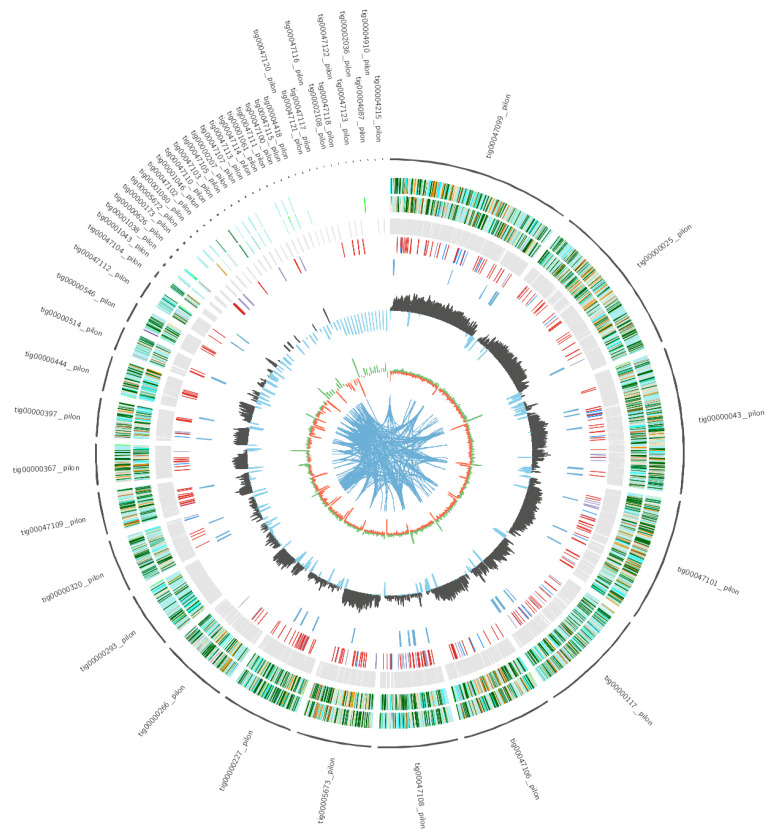
Genome diagram of strain R1.

The genome sequence of strain R1 was assembled and deposited in the NCBI GenBank database (SRA accession No. PRJNA608251) followed by a comprehensive analysis using the paired-end Illumina HiSeq 2500. The genome diagram of strain R1 shows that there are nine circles in the circle diagram (Figure 2), which are as follows from inside to outside: the first blue line shows in-paralog pairs (better hits to each other, and the evaluation between pairs is less than 1 × 10^−5^); the second circle shows the GC skew, with the green part showing a positive GC SKEW and the orange part showing a negative GC SKEW; the third circle shows the GC content; the fourth circle shows secondary metabolites; the fifth circle shows ncRNA; the sixth circle shows repeat; the seventh circle and the eighth circle display CDS annotation information, and different colors represent different COG annotation classification. The seventh circle indicates that CDS is in a negative chain, and the eighth circle indicates that CDS is in a positive chain. The outer rim shows the scaffold.

The whole-genome size of strain R1 was 51.8 Mb. This consisted of 542 contigs with an N50 of 3.21 Mb and 50.4% GC content. The results indicated that genome assembly was of high quality. A total of 58,040,324 raw reads and 54,020,630 clean reads were generated in the Illumina sequencing. We predicted 19,333 protein-coding genes, the total length of the gene was 24.69 Mb, the average sequence length was 1276.94 bp, and the longest contig length was 6.56 Mb (Table 1). We used Homology, Snap and Augustus prediction methods to predict the encoding gene. Simultaneously, the three prediction methods Proteinmask, repeatmasker and trf were used to predict repeated sequences. Proteinmask predicted that the number of repeating sequences was 1665, occupying 1.59% of the whole genome, repeatmasker predicted that the number of repeating sequences was 577, occupying 0.52% of the whole genome, trf predicted that the number of repeating sequences was 4191 occupying 0.68% of the whole genome. The number of DNA transposons was 375, occupying 0.55% of the whole genome. For non-coding RNA, we predicted 338 secondary structures of RNA and tRNA by tRNAscan, and 111 rRNA was predicted by RNAmmer. At the same time, 29 snRNA was predicted with the Rfam database by rfam_scan.

### 2.3. Genome Sequence Annotation

**Figure 3:**
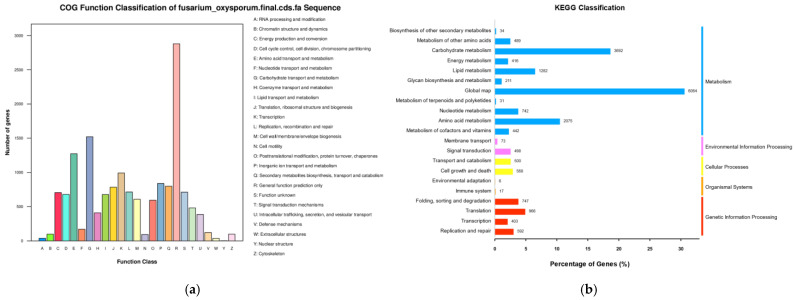

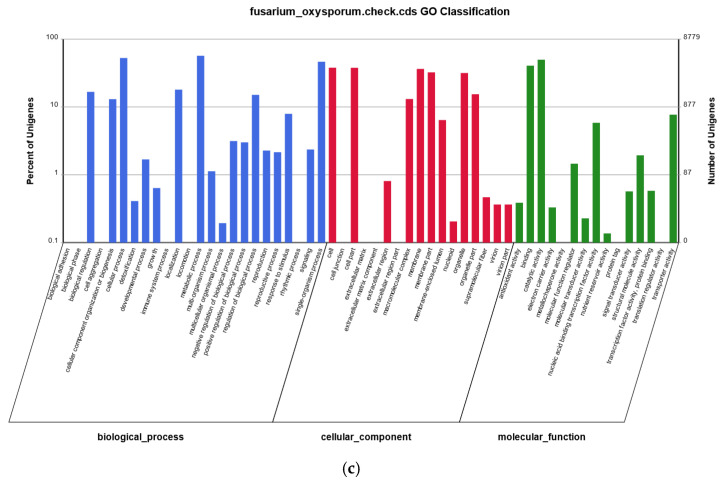
Functional annotation of strain R1 genes encoding the proteins: (**a**) Orthologous Groups of proteins (COG) analysis; (**b**) Kyoto Encyclopedia of Genes and Genomes (KEGG) analysis; (**c**) Gene Ontology (GO) analysis.

To predict protein sequences, 19,333 non-redundant genes of strain R1 were subjected to similarity analysis based on six public databases. Most genes were mapped using the Nr database (16,803 genes/86.91%), Nt (15,461 genes/79.97%), Swiss-Prot (11,026 genes/57.03%), (GO; 8780 genes/45.41%), Kyoto Encyclopedia of Genes and Genomes (KEGG; 10,894 genes/56.35%), and Clusters of Orthologous Groups (COG; 8084 genes/41.63%) (Table 1). According to the COG database, “general function prediction only” was associated with the most genes (2879) followed by “carbohydrate transport and metabolism”, “amino acid transport and metabolism”, and “transcription” as the most gene-rich classes in the COG groupings (Figure 3a) [19]. These findings suggest the presence of an enriched and varied array of carbohydrates and amino acid metabolism functions that maybe enable higher energy conversion efficiency. The KEGG functional classification showed the Global map (6064), carbohydrate metabolism (3692), and Amino acid metabolism (2075) (Figure 3b) [20]. These findings suggest the presence of an enriched and varied array of protein and lipid metabolism functions that probably enable higher secondary metabolism efficiency. GO annotation resulted in the cell (3330), membrane (3195), and organelle (2781) from the cellular component category, cellular process (4627), metabolic process (4995), and single-organism (4078) from biological processes, and binding (3567) and catalytic activity (4375) from molecular functions (Figure 3c) [21]. Strain R1 is a wild strain, in which many metabolic genes may be involved in signal transduction.

### 2.4. Additional Annotation

#### 2.4.1. Pathogen Host Interactions (PHI)

**Figure 4:**
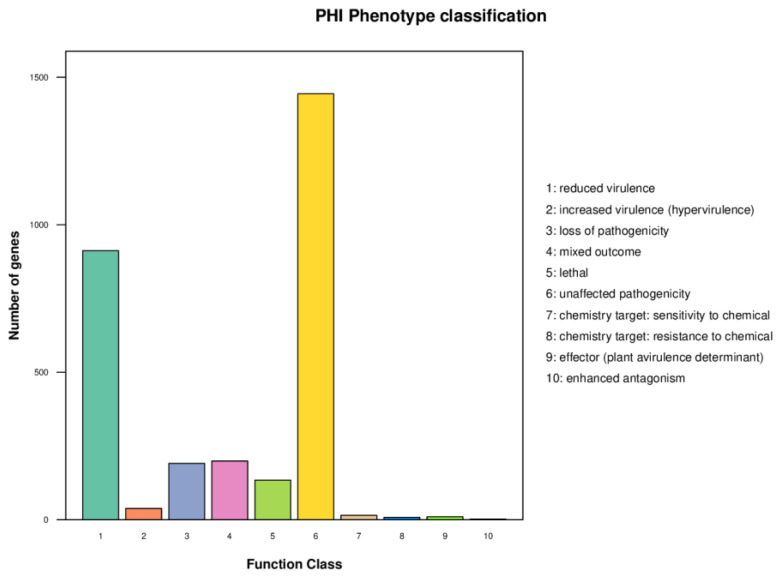
Distribution map of mutation types in the pathogen PHI phenotype of strain R1.

The Pathogen Host Interactions Database (PHI-base) has manually curated experimentally verified pathogenicity, virulence and effector genes from fungal, bacterial and protist pathogens [22]. The amino acid sequence of the target species of strain R1 was compared with the PHI database by using the BLAST software, and the gene of the target species was combined with the functional annotation information to obtain an annotation result. As shown in (Figure 4), strain R1 harbors abundant PHI-base genes, including reduced virulence (912), increased virulence (hypervirulence) (38), loss of pathogenicity (191), mixed outcome (199), lethal (134), unaffected pathogenicity (1444), sensitivity to chemical (15), resistance to chemical (8), effector (plant avirulence determinant) (10) and enhanced antagonism (2) [23]. Reduced virulence and unaffected pathogenicity are the major annotation gene, suggesting that strain R1 is not a highly pathogenic strain.

#### 2.4.2. Carbohydrate Genes

**Figure 5:**
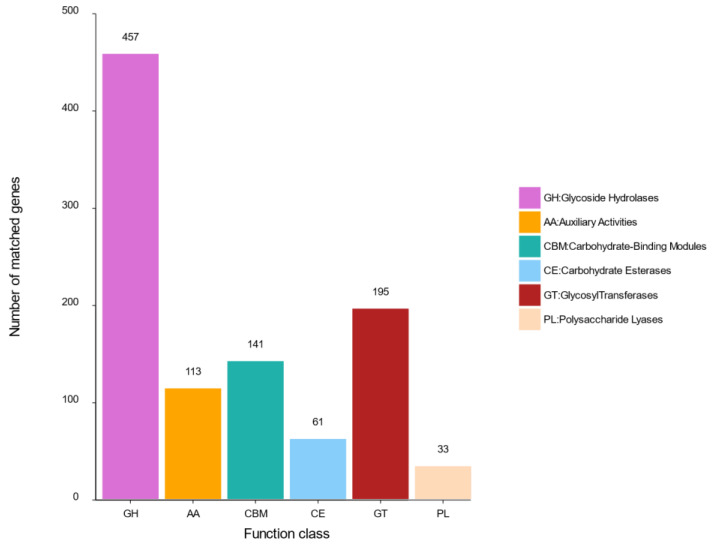
CAZy functional classification and the number of corresponding genes of strain R1.

Carbohydrate-active enzymes (CAZy) play an important role in carbohydrate degradation, modification and biosynthesis in fungi [24]. CAZy is also a Carbohydrate Active enZYmes Database, a specialized database of carbohydrate enzymes, which includes a family of related enzymes that catalyze the degradation, modification, and biosynthesis of carbohydrates [25]. This analysis showed 1000 genes encoding carbohydrate-active enzymes (CAZy) that were distributed in strain R1. These included 457 glycoside hydrolases (GHs), 113 auxiliary activities (AAs), 195 glycosyltransferases (GTs), 61 carbohydrate esterases (CEs), 141 carbohydrate-binding modules (CBMs), and 33 polysaccharide lyases (PLs) (Figure 5). PLs were mainly distributed in six families, including PL1, PL3, PL4, PL7, PL9 and PL20. AAs mainly included AA1-9, AA11-AA13 twelve families. GTs contained 29 families, including eight chitin synthetases that belong to the GT2 family. CEs were classified as CE1-CE6, CE8-9, CE11-CE12, CE16 eleven families. GHs were distributed across 65 families. Comparing with other different fungi, strain R1 had more carbohydrate genes (Table S2). Therefore, strain R1 probably has the ability to breakdown complex carbohydrates and capture more energy.

### 2.5. Analysis of Secondary Metabolite Biosynthetic Gene Clusters

**Figure 6:**
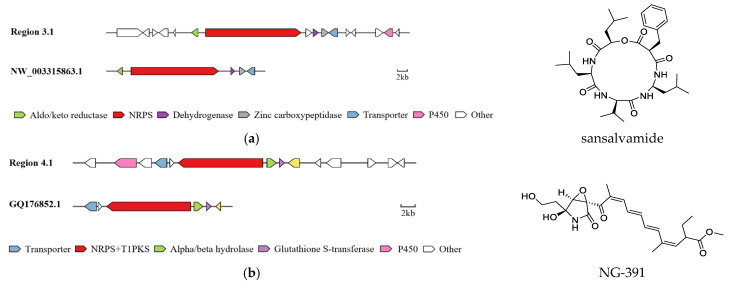

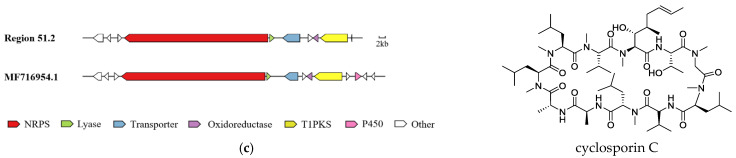
Comparison of BGC constituents in strain R1 with identified BGCs for biosynthesis of sansalvamide (**a**), NG-391 (**b**) and cyclosporin C (**c**).

Basic gene findings of its genomic sequence indicated that 18,956 genes are predicted and classified into 24 types, while 907 of these functional genes are involved in SM biosynthesis, transport and catabolism. AntiSMASH analysis suggested that strain R1 possesses 37 SM biosynthetic gene clusters (BGCs), including 13 PKS (12 T1PKS and 1 T3PKS), 10 NRPS, 7 NRPS-like, 2 hybrid NRPS + T1PKS, 1 hybrid NRPS-like + T1PKS, 1 hybrid Indole + NRPS and 3 Terpene biosynthetic genes (Table S1). Only 20% of these BGCs showed gene homologies with known clusters in the MIBiG database. By further comparison with the gene sequences of other reference strains, several BGCs of strain R1 with high similarity were identified and predicted to be responsible for the biosynthesis of sansalvamide in region 3.1, NG-391 in region 4.1 and cyclosporin in region 51.2 (Figure 6) [26,27,28].

AntiSMASH analysis showed that the genes within the region 3.1 had a significant BLAST hit with the sansalvamide BGC (GenBank: NW_003315863.1) from *F. solani* (77-13-4; FGSC 9596). Sansalvamide, a cyclic pentadepsipeptide with a potent anticancer effect, was originally isolated from one marine *Fusarium* species [29]. The chemical structure of sansalvamide, with four proteogenic amino acids and one hydroxyl acid, suggests that it could be synthesized by a five-module NRPS where each of the modules would be responsible for incorporating one of the amino acids. BGC region 4.1 of strain R1 displayed significant similarity with that of NG-391 (GenBank: GQ176852.1) from *Metarhizium robertsii*. NG-391 is the 7-desmethyl analog of fusarin C, and like fusarin C, NG-391 is strongly mutagenic in the Ames test in the presence of the S9 fraction from rat liver [30,31,32]. Given its structural similarity to fusarin C, NG-391 is expected to be produced by a hybrid PKS–NRPS using a similar biosynthetic mechanism. Typically, these megasynthases combine a type I PKS with a single NRPS module and a C-terminal reductase domain. In addition to six ORFs, BGC region 4.1 possesses several additional enzymes including an aminotransferase class V, one cytochrome P450, and aldehyde dehydrogenase. BGC region 51.2 of strain R1 showed a highly similar sequence with the cyclosporins C BGC (GenBank: MF716954.1) from *Beauveria feline*. Cyclosporin C belongs to one class of cyclic depsipeptides and is used as a cyclophilin inhibitor for the prevention or treatment of diseases or disorders, such as organ injury or organ failure [33,34]. Thirteen genes involved in the biosynthesis of cyclosporin C were highly similar (identities and similarities > 80%) with the BGC of CsA from *Tolypocladium inflatum* NRRL 8004. Few fungi except *Aspergillus terreus* [35] had been reported to make cyclosporin agents, while others (*Leptostroma*, *Cylindrotrichum*, *Stachybotrys*) produce novel cyclosporin analogs [36]. The high similarity of these genes indicates that strain R1 has the capability to synthesize these motif-containing SMs. Furthermore, additional genes encoding P450 enzyme, dehydrogenase and protease in these BGCs endow this strain with the potential to biosynthesize more novel compounds. Additionally, 13 BGCs are predicted to code for single PKSs including 12 T1PKS and 1 T3PKS. However, only 3 PKSs located in regions 1.2, 53.1 and 72.1 displayed low similarities with known clusters responsible for the biosynthesis of duclauxin (GenBank accession no. EQ962653.1) [37], gibepyrone-A BGC (GenBank accession no. HF679033.1), oxyjavanicin (GenBank accession no. HE613440.1), respectively [38]. The function of other cryptic BGCs need to be further characterized by gene knockout experiment and heterogeneous expression, as well as LC-MS analysis.

**Figure 7:**
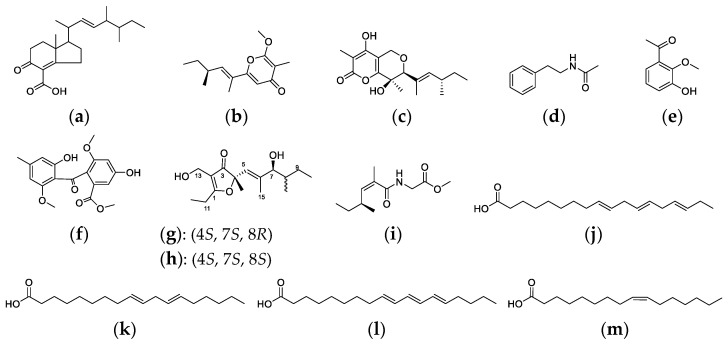
Thirteen secondary metabolites (**a**–**m**) previously discovered from strain R1.

In our previous chemical investigation of strain R1, two novel polyketides (Figure 7a,b) along with eleven known substances (Figure 7c–m) had been isolated and characterized (Figure 7) [39,40,41]. Antimicrobial tests showed that compound **1** had a potent inhibitory effect on *Staphyloccocus aureus* ATCC 2592 with a MIC value of 6.25 μM and compound **6** displayed weak anti-*Helicobacter pylori* capability at 16 μM. On basis of the antiSMASH analysis, compounds **a**–**c**, **e**–**h** and **j**–**m** were putatively biosynthesized by various PKSs, while compounds **d** and **i** were plausible products of the hybrid NRPS + PKS [42].

## 3. Materials and Methods

### 3.1. Microbes and Cultivation

The endophytic strain R1 was isolated and purified from the coastal plant *Rumex madaio* Makino, collected off Putuo Island (Zhoushan, China) [43]. A suspension of culture containing its mycelia in PDA supplemented with glycerol (20% *v*/*v*) was stored at −80 °C at Zhejiang University of Technology (Hangzhou, China).

### 3.2. Phylogenetic Analysis

For phylogenetic analysis, strain R1 was cultivated in a PDB medium at 28 °C for 3 days followed by *18S* rRNA and ITS gene amplicon sequencing. Both the *18S* rRNA sequence (GenBank accession no. MF376147) and ITS sequence (GenBank accession no. ON545070) were submitted to the NCBI databases. The phylogenetic analysis of strain R1 was delineated by a neighbor-joining phylogenetic tree which was constructed using the Tamura3-parameter model in MEGA7 with 1000 bootstrap replicates [44].

### 3.3. Genome Sequencing and Assembly

Strain R1 was grown on a PDA medium for 7 days at 28 °C. Genomic DNA was extracted following the CTAB extraction protocol, the concentration was verified fluorometrically using Gentra Puregene Yeast/Bact. Kit (Qiagen, Valencia, CA), the integrity and purity were assessed by 1% agarose gel electrophoresis and Nanodrop2000, and then dissolved in sterile water and adjusted to a concentration of 149 ng/μL. The 18*S* rRNA gene was amplified by PCR using the universal primers NS1 (5′-GTAGTCATATGCTTGTCTC-3′) and NS6 (5′-GCATCACAGACCTGTTATTGCCTC-3′). The PCR conditions included an initial denaturation at 94 °C for 15 min, followed by 30 cycles of 45 s at 94 °C, 90 s at 55 °C and 90 s at 72 °C; and a final extension at 72 °C for 10 min. Then to build and check the library, DNA libraries of a certain concentration and volume were added to each independent Flow cell, and the Flow cell was transferred to GridION X5 sequencer (Nanopore, Oxford, UK) for real-time single-molecule sequencing. Qubit (v2.0) was used for initial quantification, then the insert size of the library was detected using Agilent 2100, in order to ensure the quality of the library, Q-PCR was used to quantify the library’s effective concentration. The third-generation sequencing reads were assembled by HGAP (v4, http://www.pacb.com/devnet/, accessed on 10 December 2021) [45] and CANU (v1.7.1, https://canu.readthedocs.io/en/latest/, accessed on 20 December 2021) [46] software into contigs. The qualified library was sequenced by the paired-end Illumina HiSeq 2500, and the Raw Data obtained from the sequencing was used for post-processing information analysis. After the sequencing of genomic DNA, the Paired-end raw data were saved in FASTQ format. Quality control on Paired-end raw reads from next-generation sequencing data was performed using FastQC, the 3′ end of DNA adapter contamination was decontaminated with Adapter Removal protocol. At this point, raw data were filtered for high-quality adapter-free reads for genome assembly. For corrected read assembly, Unicycler (https://github.com/rrwick/Unicycler, accessed on 15 February 2022) was used to finalize the optimal assembly. Finally, Pilon software (v1.18, https://github.com/broadinstitute/pilon, accessed on 21 February 2022) was utilized to correct the third-generation contigs with the above-mentioned high-quality next-generation sequencing data and stitch them together to assemble a complete strain R1 genome sequence [47].

### 3.4. Gene Prediction and Annotation

Gene prediction was performed using Homology, SNAP and Augustus. Based on the gene function and metabolic pathway of the existing databases, the function annotation was performed by BLAST searches against these databases: NR (NCBI non-redundant protein sequences), Swiss-Prot, KEGG (Kyoto Encyclopedia of Genes and Genomes), COG (Cluster of Orthologous Groups of proteins), PHI (Pathogen Host Interactions Database), CAZy (Carbohydrate-Active Enzymes Database).

### 3.5. Analysis of Secondary Metabolite Biosynthetic Gene Clusters

SM biosynthetic gene cluster analysis of strain R1 was carried out by antiSMASH fungal 6.0.1 [48]. AntiSMASH can accurately identify all known secondary metabolic gene clusters when it can use a specific profile hidden Markov models [49]. In order to further study the obtained gene clusters, we used the NCBI Genome Portal Software Platform to conduct Blastp analysis and gene annotation, and then, concluded the gene clusters of secondary metabolites in strain R1.

## 4. Conclusions

*Fusarium* is a treasure trove of SMs with diverse chemical structures and biological properties [50]. In addition to phylogenetic analysis based on the *18S* rRNA gene sequence, a high-quality whole-genome sequence of endophytic strain *Fusarium* sp. R1 from *R*. *madaio* Makino was obtained and extensively analyzed by gene prediction and annotation in this work. The results showed that strain R1 harbors abundant functional genes in energy production and conversion, amino acid transport and metabolism, carbohydrate transport and metabolism, secondary metabolites biosynthesis, transport and catabolism.

AntiSMASH analysis of strain R1 uncovered only 8 of 37 BGCs showed high similarity with known gene clusters, suggesting it had a vast potential for producing other SMs. Our previous chemical investigation indicated that most SMs produced by strain R1 under normal conditions are polyketides. Therefore, a great number of other types of BGCs of this strain are silent and/or expressed at a low level. These findings open possibilities for targeted genome mining such as gene knockout, introduction or heterologous expression of microbial genes, regulation of promoters, and induction of mutations to awaken these silent BGCs to biosynthesize more new bioactive SMs for new drug research and development [51].

## Figures and Tables

**Table 1:** Genomic assembly and functional annotation of strain R1 genome.

Item	Value	Item	Count	Percentage (%)
Total length (bp)	51,784,516	All	17,145	88.68
Max length (bp)	6,563,362	NR	16,803	86.91
GC content (%)	50.4	NT	15,461	79.97
Gene number	19,333	Swiss-Prot	11,026	57.03
Gene total length (bp)	24,687,144	KEGG	10,894	56.35
Gene average length (bp)	1276.94	COG	8048	41.63
GC content in gene region (%)	55.06	GO	8780	45.41
Gene/Genome (%)	47.67			
Contigs	542			
N50 (bp)	3,209,824			
N90 (bp)	1,367,080			

## Data Availability

The data presented in this study are available in supplementary material.

## Supplementary Materials

The following supporting information can be downloaded at: https://www.mdpi.com/article/10.3390/metabo12060521/s1, Table S1: Putative biosynthetic gene clusters (BGCs) coding for secondary metabolites in strain R1. Table S2. Gene distribution of different fungi based on the six major modules of CAZymes. Figure S1. Phylogenetic tree of strain R1 based on *18S* rRNA gene sequences aligned in NCBI standard database. Figure S2. Phylogenetic tree of strain R1 based on ITS gene sequences aligned in NCBI rRNA/ITS database.
