# Supplementary material for: Genome Features and AntiSMASH Analysis of an Endophytic Strain Fusarium sp. R1
Source: Metabolites. 2022 Jun 4;12(6):521. doi: 10.3390/metabo12060521 (PMC9229708; doi:10.3390/metabo12060521)
Supplement: Supplementary file 1 [file metabolites-12-00521-s001.zip › metabolites-1698445-supplementary.pdf]

Supplementary materials for

Genome Features and antiSMASH Analysis of an Endophytic Strain *Fusarium* sp. R1

Yuanyuan Liu†, Meijie Xu†, Yuqi Tang, Yilan Shao, Hong Wang and Huawei Zhang*

School of Pharmaceutical Sciences, Zhejiang University of Technology, Hangzhou 310014, China

* Correspondence: hwzhang@zjut.edu.cn; Tel.: +86-571-88320913
† These authors contributed equally to this work.

Table S1. Putative biosynthetic gene clusters (BGCs) coding for secondary metabolites in strain R1.

BGCs	From	To	Type	Most similar known cluster (%)
Region 3.1	153,629	210,853	NRPS	Sansalvamide(100%)
Region 3.2	907,120	963,948	NRPS	Unknown
Region 13.1	279,194	323,633	NRPS	Unknown
Region 15.1	757,738	803,168	NRPS	Unknown
Region 16.2	438,749	485,036	NRPS	Unknown
Region 36.1	135,448	191,933	NRPS	Unknown
Region 45.1	34,875	80,743	NRPS	Dimethylcoprogen(100%)
Region 53.2	152,336	200,243	NRPS	Unknown
Region 111.1	1	44,042	NRPS	Unknown
Region 219.1	1	11,588	NRPS	Unknown
Region 1.1	136,984	180,346	NRPS-like	Unknown
Region 2.1	1,695,442	1,739,344	NRPS-like	Unknown
Region 16.1	26,326	67,542	NRPS-like	Unknown
Region 28.1	550,037	593,921	NRPS-like	Unknown
Region 54.1	95,280	139,642	NRPS-like	Unknown
Region 89.1	51,173	94,979	NRPS-like	Unknown
Region 114.1	32,992	61,293	NRPS-like	Unknown
Region 61.1	14,358	84,228	NRPS, Indole	Unknown
Region 4.1	943,332	995,218	NRPS, T1PKS	NG-391 (100%)
Region 51.2	223,941	318,761	NRPS, T1PKS	Cyclosporin C (69%)
Region 3.4	1,198,909	1,253,104	NRPS-like, T1PKS	Unknown
Region 1.2	1,222,174	1,268,436	T1PKS	Duclauxin(35%)
Region 3.3	973,808	1,015,241	T1PKS	Unknown
Region 10.1	429,817	477,393	T1PKS	Unknown
Region 12.1	614,221	661,232	T1PKS	Unknown
Region 20.1	470,252	520,164	T1PKS	Unknown
Region 22.1	356,352	404,197	T1PKS	Unknown
Region 51.1	2,962	48,533	T1PKS	Unknown
Region 53.1	31,031	79,330	T1PKS	Gibepyron-A (40%)
Region 66.1	143,009	190,982	T1PKS	Unknown
Region 72.1	131,149	171,004	T1PKS	Oxyjavanicin(37%)
Region 93.1	57,933	102,230	T1PKS	Unknown
Region 192.1	1	17,285	T1PKS	Unknown
Region 13.2	520,314	561,786	T3PKS	Unknown
Region 5.1	1,032,138	1,053,178	Terpene	Unknown
Region 6.1	524,894	546,441	Terpene	Squalestatin(40%)
Region 18.1	375,397	396,599	Terpene	Unknown

Table S2. Gene distribution of different fungi based on the six major modules of CAZymes.

CAZy	Total	GH	AA	GT	CBM	CE	PL
Fusarium sp. R1	1000	457	113	195	141	61	33
Inonotus obliquus	380	199	76	66	11	24	13
Aspergillus nidulans	486	259	89	81	20	32	23
Aspergillus niger	503	253	105	102	17	30	10
Aspergillus oryzae	538	289	96	93	17	31	26
Gloeophyllum trabeum	350	199	57	62	5	19	11
Trametes versicolor	441	223	106	78	6	19	13
Coprinopsis cinerea	469	188	129	72	21	47	18
Schizophyllum commune	469	247	85	71	11	37	19
Pleurotus ostreatus	505	226	139	65	29	27	26

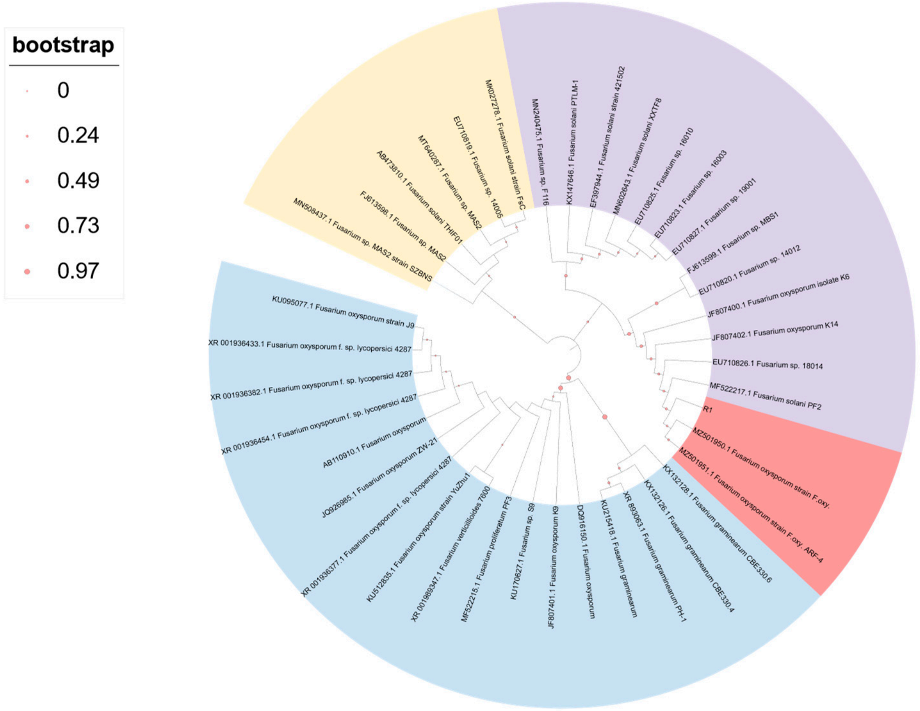

Figure S1. Phylogenetic tree of strain R1 based on *18S* rRNA gene sequences aligned in NCBI standard database.

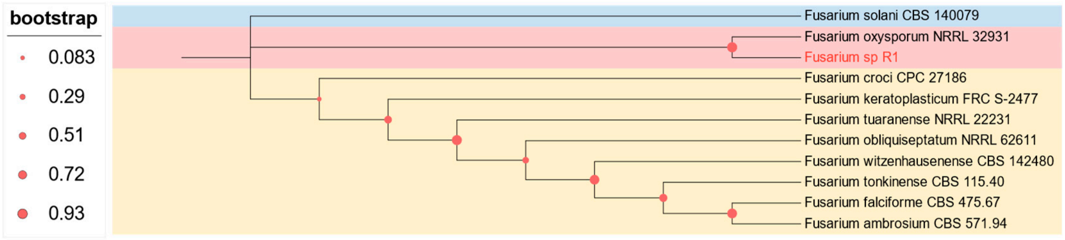

Figure S2. Phylogenetic tree of strain R1 based on ITS gene sequences aligned in NCBI rRNA/ITS database.
